# Mathematical Modeling of “Chronic” Infectious Diseases: Unpacking the Black Box

**DOI:** 10.1093/ofid/ofx172

**Published:** 2017-08-14

**Authors:** Anthony T Fojo, Emily A Kendall, Parastu Kasaie, Sourya Shrestha, Thomas A Louis, David W Dowdy

**Affiliations:** 1 Division of General Internal Medicine; 2 Division of Infectious Diseases, Johns Hopkins University School of Medicine, Baltimore, Maryland; 3 Department of Epidemiology; 4 Department of Biostatistics, Johns Hopkins Bloomberg School of Public Health, Baltimore, Maryland

**Keywords:** tuberculosis, HIV, hepatitis C, theoretical models, Bayesian analysis

## Abstract

**Background:**

Mathematical models are increasingly used to understand the dynamics of infectious diseases, including “chronic” infections with long generation times. Such models include features that are obscure to most clinicians and decision-makers.

**Methods:**

Using a model of a hypothetical active case-finding intervention for tuberculosis in India as an example, we illustrate the effects on model results of different choices for model structure, input parameters, and calibration process.

**Results:**

Using the same underlying data, different transmission models produced different estimates of the projected intervention impact on tuberculosis incidence by 2030 with different corresponding uncertainty ranges. We illustrate the reasons for these differences and present a simple guide for clinicians and decision-makers to evaluate models of infectious diseases.

**Conclusions:**

Mathematical models of chronic infectious diseases must be understood to properly inform policy decisions. Improved communication between modelers and consumers is critical if model results are to improve the health of populations.

Mathematical models represent the mechanisms and temporal dynamics of infectious disease epidemics and are increasingly used in the field of infectious diseases to better understand natural history, make predictions about the future, and evaluate potential interventions [1–3]. As modeling studies have proliferated and grown more complex, it has become more difficult for clinicians and policy makers to understand their inner workings [1, 3]. Interpretation of these models’ conclusions without an understanding of their underlying assumptions and mechanics can be misleading [4].

The infectious diseases studied with such models may be classified as either acute infections, such as cholera [5], measles [6], and Ebola [4], or “chronic” infections with a longer average generation time between infection and subsequent transmission, such as HIV [7], tuberculosis (TB) [8], and hepatitis C [9]. We focus on mechanistic models of chronic infectious diseases and highlight how different design choices can lead models to different conclusions and uncertainty estimates, even when using the same data, with implications for policy decisions. Our primary aim is not to describe the principles of infectious disease modeling (which can be found elsewhere [2, 10, 11]), but rather to make models of chronic infections more understandable—and thus more useful—to clinicians and policy makers.

## MOTIVATING EXAMPLE

For the purposes of illustration, we evaluate the potential impact of a hypothetical active case-finding intervention for TB, measured in terms of percent reduction in projected TB incidence by 2030, compared against the existing standard of care. We assume that this hypothetical intervention reduces the average time to diagnosis of infectious TB by 25% and use India as an illustrative setting. Since this is a hypothetical intervention, the results presented are meant to be demonstrative only. India has a high incidence of TB (217 per 100 000 in 2016) [12], and active case-finding (an attempt to diagnose people with TB before they would otherwise present to care) has been prioritized by the World Health Organization (WHO) and other policy-making bodies as a potential means to reduce TB transmission [13].

**Figure 1.**
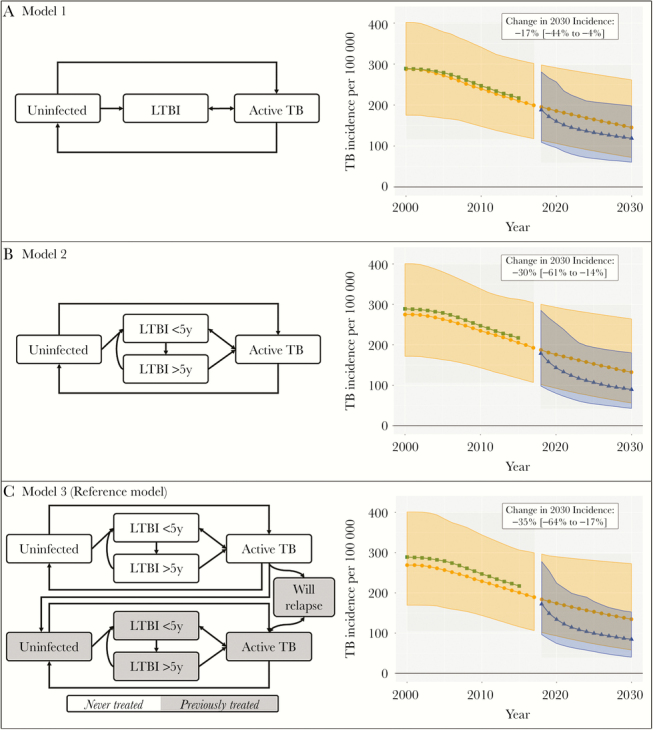
The effect of model structure on results. Each panel presents a model structure adjacent to a graphical representation of the fit of that model. The dark squares indicate the World Health Organization incidence estimates (the same for all models). The light circles and ribbons indicate model estimates of tuberculosis (TB) incidence and 95% uncertainty intervals in the absence of the intervention. The dark triangles and ribbons indicate model estimates of TB incidence and 95% uncertainty intervals in the presence of the intervention. The white inset indicates the estimate and 95% uncertainty interval of our primary outcome: the change in 2030 TB incidence with vs without the intervention. In addition to the structural elements depicted in the figures, each model also assumes that the transmission rates of tuberculosis decline linearly from 2002 to 2030. Latent TB infection (LTBI) < 5 years denotes a latent infection acquired within the past 5 years. LTBI > 5 years denotes a latent infection acquired more than 5 years earlier.

To evaluate this intervention, we model the existing TB epidemic in India and project that epidemic forward in time, comparing a scenario with the hypothetical case-finding intervention against an otherwise identical scenario in which the intervention is not employed. Results from 3 models are shown in Figure 1. Prior to 2015, the model estimates (light circles) are compared with WHO estimates of TB incidence in India (dark squares). Beyond 2015, model projections without the intervention (light circles) are compared with the intervention projections (dark triangles) in order to estimate the primary outcome (difference in TB incidence by 2030), as well as the attendant uncertainty. For example, the model in panel C (the reference model) projects a change of –35% in incidence (95% uncertainty interval, –65% to –17%) due to the intervention. In the following sections, we will explore the assumptions and design choices that undergird these results.

## MODELS AND SOURCES OF UNCERTAINTY

Models of infectious diseases grapple with uncertainties in the parameters that drive epidemiological dynamics and in the observations used to inform the models. Even the best models cannot capture all possible fluctuations in transmission dynamics, and so are subject to further uncertainty. Models may be deterministic—one set of parameters always producing the same result—or stochastic—with an element of randomness, such that running the model twice with the same parameters produces different results. Stochastic models additionally entail uncertainty engendered by the randomness in the model.

Bayesian approaches offer an effective way to manage these uncertainties [14–16]. There are a number of different Bayesian techniques; all run the model many times with a range of different parameters to generate a set of simulations. The probability of a simulation contributing to the final result is proportional to how well the simulation fits the observed epidemic. The set of epidemic trajectories consistent with observed data can be used to estimate specific outcomes and the uncertainty around them.

In addition to quantifying uncertainty, effective modeling work also examines the key drivers of results through sensitivity analyses. In the following sections, we describe the components of a Bayesian approach and use a deterministic transmission model to highlight how incorrect handling of uncertainty could cause results to be misleading and potentially undermine policy decisions. In our technical supplement, we present the mathematical underpinnings of these techniques in greater depth.

## MODEL STRUCTURE

Structuring a model involves a trade-off between simplicity and fidelity: a more complex model may represent reality more completely, but each added element requires additional data that may not be available or that carry their own uncertainty [1]. In general, one strives for a model that includes all features relevant to the research question without unnecessary complexity [2].

In Figure 1, we present 3 models. The first and simplest (panel A) represents the population as either susceptible, latently infected with TB, or having active (infectious) TB. The second (panel B) introduces a second latent state (after 5 years), which allows rates of reactivation to be higher for more recent infections. The third (panel C) stratifies the population by previous treatment status, allowing us to explicitly model relapse of treated TB and lower treatment success among those who have been previously treated.

Depending on the research question, we could make our model more complex—for example, by incorporating age, HIV, or drug resistance. Added complexity is beneficial if it improves the model’s ability to answer the study question [17], but overly complex models may reduce transparency or replicability and may require more parameter estimates than exist data to accurately inform. In our motivating example, an explicit representation of resistance to second-line TB drugs (given a question involving mostly drug-susceptible TB) likely would not justify the additional uncertainty.

Figure 1 shows that the primary outcome—the projected change in TB incidence by 2030 from the case-finding intervention—is greater in models 2 (–30%, 95% uncertainty interval [UI], –61 to –14%) and 3 (–35%, 95% UI, –64 to –17%) than in model 1 (–17%, 95% UI, –44 to –4%). This reflects the nuance that models 2 and 3 estimate a higher proportion of active TB resulting from recent infection, and reductions in transmission via faster case-finding have more immediate impact on incidence. Ignoring the fact that progression to active TB is more common soon after infection leads model 1 to underestimate the impact of our intervention. While no model is a perfect representation of reality, this illustration highlights that it is critical to consider the uncertainty engendered by the choice of model structure. For example, if comparing to another intervention projected to reduce TB incidence by 25% over the same time period, policy makers might make different decisions about whether to implement active case-finding if the projected impact of case finding was to reduce incidence by 17% vs 35%.

Evaluating model structure depends foremost on an understanding of the biological and social processes driving an epidemic. Sometimes, more than one model is plausible. In such cases, competing models can be scored based on how likely simulations from each model are given the observed epidemic. These scores can be used either to choose the most probable model (model selection) or to combine the results from multiple models by taking a weighted average (model averaging) [18, 19]. For the purposes of our discussion, we continue subsequent analyses with model 3 because our scientific understanding indicates that prior treatment is relevant to future TB.

## SAMPLING INPUT PARAMETERS

If the model structure represents our qualitative understanding of the processes important to our research question, the associated input parameters are our attempt to quantify, using available data, the processes we have chosen to represent. These data are inherently uncertain: published estimates represent studies of limited size and generalizability.

Because we intend for models to provide both point estimates and characterizations of uncertainty, we specify a “best guess” for each parameter and a probability distribution (“prior distribution”) reflecting our understanding of the parameter’s underlying uncertainty. Our final results will be influenced by the prior distributions that we specify, which are characterized by a range and a shape: wider ranges and flatter shapes suggest greater uncertainty. The flattest shape is a uniform distribution, where all values in the range are equally likely. Distributions that have central tendency—such as normal or log-normal distributions—indicate a belief that the true parameter value is more likely near the central value than at the edges of the range.

Consider the parameter for long-term reactivation risk (more than 5 years after infection). One reasonable estimate for this parameter comes from comparing observed case rates to a population-based tuberculin skin test survey in Florida [20]. The study reported high and low estimates for the reactivation rate, each with a confidence interval. We could construct the prior for this parameter as a uniform distribution between the lower bound for the lower estimate and the upper bound for the higher estimate. Alternatively, we could choose a narrower range or a distribution with a central peak—but this might overstate our confidence in the applicability of results from a small study in Florida to our target population in India. In general, some parameters—such as this reactivation rate—will be relatively uncertain and merit wider, flatter distributions. Others—such as treatment success, which is directly measured in India—will have less uncertainty, meriting narrower, more peaked distributions.

**Figure 2.**
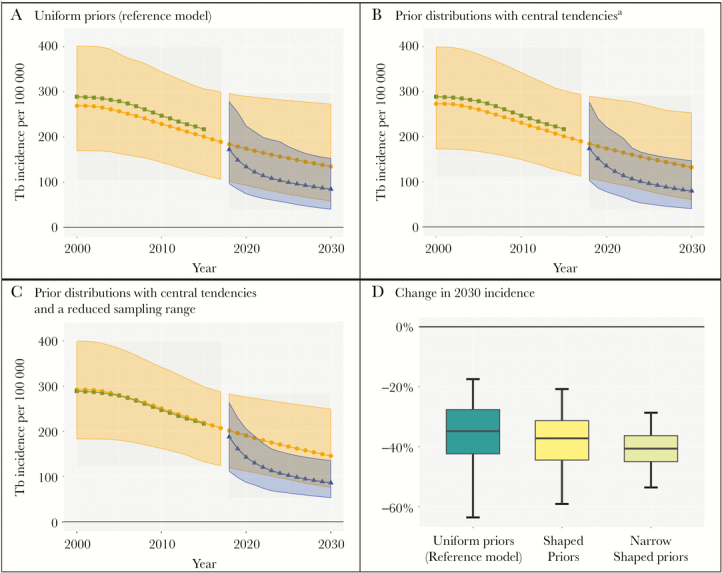
The effect of prior distributions on results. Panels A, B, and C present graphical representations of the model fit under different formulations of prior distributions. Panel D presents a box-and-whiskers plot for the primary outcome under each set of priors: the dark horizontal line indicates the estimate, the shaded box indicates the 50% uncertainty interval, and the whiskers indicate the 95% uncertainty interval. ^a^Prior distributions with central tendencies are either log-normal distributions when the parameter is a rate (with possible values 0 to infinity) or logit-normal distributions when the parameter is a proportion (with possible values 0 to 1). The standard deviations of these distributions are chosen so that the sampling range corresponds to a 95% confidence interval. TB, tuberculosis.

**Figure 3.**
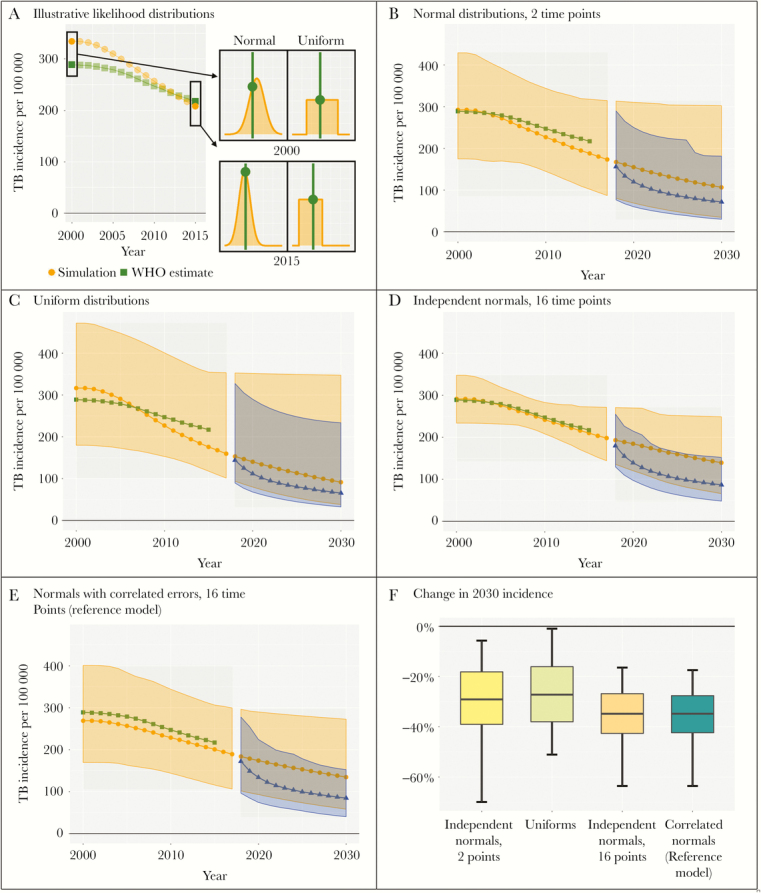
The effect of likelihood on results. Panel A compares the use of 2 distributions—normal and uniform—in the likelihood at years 2000 and 2015.The light circles represent a sample simulation, while the dark squares represent the World Health Organization (WHO) estimates. The curves on the right represent the distributions for those years, and the value of the likelihood is the vertical distance to the intersection of the curve and the WHO estimate, indicated by the green circle. The normal likelihood gives greater weight when the simulation estimate is closer to the WHO estimate, while the uniform likelihood gives equal weight to any estimate that falls within the WHO range. The next 4 panels present graphical representations of the model fit using a likelihood made of (B) normal distributions at 2000 and 2015, (C) uniform distributions on each of the 16 years from 2000 to 2015, (D) independent normal distributions on the 16 years from 2000 to 2015, and (E) a normal likelihood for the 16 years from 2000 to 2015, with a correlation coefficient of 0.5 between the errors in any 2 years. Panel F shows a box-and-whiskers plot for the primary outcome under each formulation of the likelihood: the dark horizontal line indicates the estimate, the shaded box indicates the 50% uncertainty interval, and the whiskers indicate the 95% uncertainty interval. TB, tuberculosis.

Figure 2 presents results using 3 models with the same structure and different prior distributions for parameters. The first (Figure 2 A), assumes a uniform distribution for all parameters over ranges derived from the literature (given in Table S1 in the technical supplement [12, 20–27]). The second (Figure 2 B), assumes prior distributions with a central tendency (log-normal or logit-normal, depending on the type of parameter). The third (Figure 3 C) also assumes peaked distributions, but cuts their sampling ranges in half. The estimate of the primary outcome is similar in all 3 models (between –40% and –35%) (Figure 1 D), but the corresponding uncertainty intervals differ substantially. The uniform prior distributions have the widest uncertainty (–64% to –17%) as those distributions suggest the least confidence in the parameter values. Assuming priors with a central tendency on the full range results in a narrower uncertainty interval (–59% to –21%), and reducing the sampling ranges further reduces the uncertainty interval to half as wide as for the uniform priors (–54% to –29%) despite using the same model structure and point estimates for all input parameters.

Choosing the range and shape of prior distributions is complex and context-dependent, with justifications generally appearing in technical appendices. In a model with a large number of parameters, it can be time consuming for the reader to evaluate the prior for each parameter individually; therefore, in the absence of a strong rationale for a unique approach to a specific parameter, it can be helpful if authors employ a principled, data-driven, a priori approach. An example of such an approach would be to use one type of distribution (eg, log-normal) for all similar types of parameters (eg, rates vs proportions), with a consistent approach to selecting ranges (eg ±25% from the point estimate) that are not too narrow—unless data exist to support deviation from this approach for a specific parameter. Overstating certainty in parameter values—whether by choosing overly narrow ranges or distributions with an overly strong central tendency—can lead to inappropriately precise projections. For example, if policy makers wanted to be relatively certain that a case-finding intervention could reduce TB incidence by 20% before enacting it as policy, a lower uncertainty bound of 29% vs 17% could make the difference between a “go” and a “no-go” decision.

## CALIBRATING THE MODEL

In a Bayesian framework, calibration of the model is the process of generating a set of simulations consistent with the observed epidemic. This entails choosing measures that represent the “real world” and weighting simulations based on how well they replicate those targets. In our example, we weight simulations according to how well they match WHO estimates of TB incidence and mortality in India from 2000 to 2015. The weight for each simulation is given by a likelihood function: the probability of observing data given a model structure and parameter values. For a deterministic model like ours, the likelihood function principally captures uncertainty pertaining to parameter values and calibration targets. For stochastic models, the likelihood additionally captures uncertainty engendered by the randomness in the process of using parameters to run a simulation. In the subsequent discussion, we focus on our deterministic model and defer consideration of process-based uncertainty to other published works [28, 29].

The likelihood should reflect the way in which the calibration targets were derived. Such targets may be directly measured or estimated indirectly. For example, not all TB cases in India are reported, so WHO estimates TB incidence using a statistical model based on more limited sampling, with uncertainty ranges that reflect potential sampling error [12, 30]. Knowledge of the process that generated the estimates yields the insight that they are likely correlated: errors in the estimated incidence are likely to be similar from year to year because they were derived in the same way.

Figure 3 illustrates the importance of the likelihood by comparing results of 4 models that all calibrate to the WHO-estimated incidence of TB from 2000 to 2015 using the same model structure, prior parameter distributions, and point estimates of incidence. These models differ only in their likelihood functions (panel A). The first model (panel B) uses normal distributions in 2000 and 2015 (giving greater weight to simulations in which WHO estimates are closer to simulated values), while the second model (panel C) uses uniform distributions (all simulations receive equal weight, as long as their estimates of incidence are within the specified range of the WHO estimates). While the estimates for both incidence and the primary outcome (panel F) in these 2 studies are similar, the uncertainty intervals are much narrower when using the normal likelihoods.

In addition to specifying the shape of the likelihood function, models must also account for potential correlation between calibration targets. The model in panel D uses a normal distribution for each of the 16 years from 2000 to 2015 and assumes that the WHO estimates for each year are independent, generating much narrower uncertainty intervals around the estimated incidence for each year. The model in panel E also uses normal distributions from 2000 to 2015, but assumes that the measurement errors in these years are correlated—resulting in wider uncertainty intervals for estimated yearly incidence. Both likelihoods generate similar ranges for the primary outcome (panel F).

**Figure 4.**
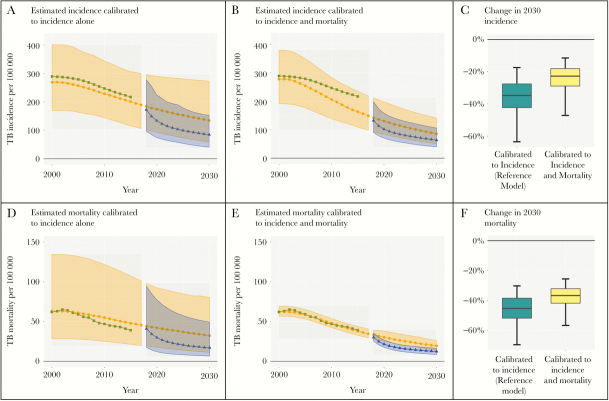
The effect of calibration targets on results. Panels A and D display the ability to reproduce incidence and mortality trends, respectively, of a model calibrated to incidence alone (using a correlated error likelihood). Panels B and E display the ability to reproduce the incidence and mortality, respectively, of a model calibrated to both incidence and mortality. Panels C and F show the changes by 2030 in incidence and mortality, respectively, if the intervention is undertaken under each of the 2 calibration procedures: the dark horizontal line indicates the estimate, the shaded box indicates the 50% uncertainty interval, and the whiskers indicate the 95% uncertainty interval. TB, tuberculosis.

Likelihood functions can incorporate more than one type of calibration target. Figure 4 illustrates the effect of calibrating to both TB incidence and TB-specific mortality. This induces a tradeoff: we fit mortality better at the expense of a worse fit to incidence. As incidence is likely more important than mortality for a case-finding intervention, calibrating to mortality may bias our estimates of future incidence. The magnitude of the trade-off suggests that our model is mis-specified with respect to mortality; if we wanted to study effects on mortality, a more complex model structure would likely be justified. In general, calibration should focus on targets that have bearing on the research question at hand, and a poor fit to relevant calibration targets should raise concern about mis-specification of the model.

While studies usually explicitly state their calibration targets, most relegate the structure of the likelihood function to the technical supplement and give little, if any, consideration to the handling of correlated measurements. However, it is important to assess whether the calibration process accurately manages uncertainty. Over-representing the level of confidence in a calibration target or treating repeated measurements as independent can lead to inappropriately precise study results. For example, if enacting a policy depended on the chances that TB incidence could reach 250 per 100 000 per year by 2030, the analysis in Figure 3D, which concludes that there is a statistically negligible probability of TB incidence reaching this threshold absent any intervention, might support a different policy than the analysis in Figure 3E, which finds such an incidence to be well within the realm of statistical possibility.

## SENSITIVITY ANALYSES

In infectious disease models, sensitivity analysis traditionally focuses on the degree to which a change in input parameters (one at a time or simultaneously) changes the primary outcomes [31, 32]. This can help to focus critical evaluation of parameter choices; if a parameter has little impact on the study results, its correct specification matters less. Sensitivity analyses can also evaluate how a model’s structure impacts the results [1].

**Figure 5.**
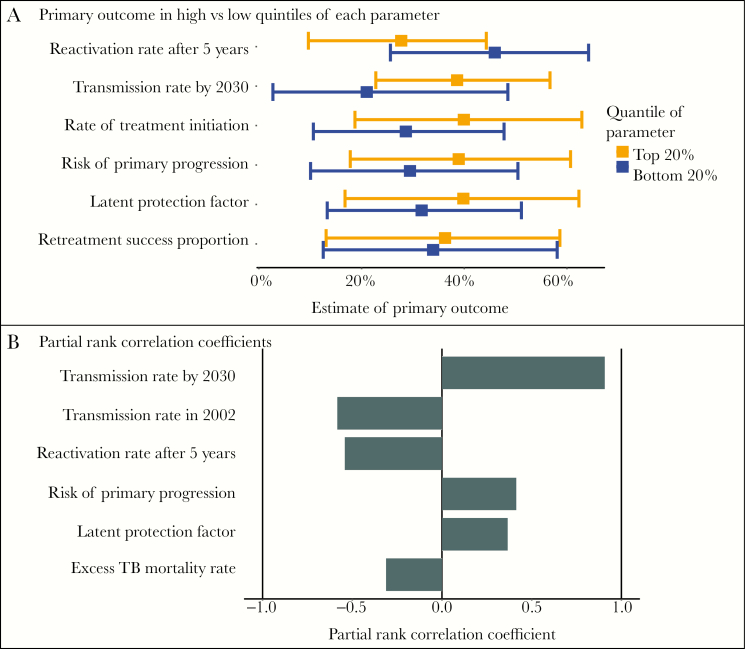
Sensitivity analyses. Panel A shows a comparison of the primary outcome (relative reduction in 2030 tuberculosis [TB] incidence if the intervention is undertaken) in high vs low quintiles of each input parameter. For each parameter, the light circle and bar show the results (estimate and 95% uncertainty interval) if we restrict our analysis to those simulations where the parameter value is among the highest 20% of all sampled values for that parameter; the dark square and bar show the results for simulations where the parameter value is in the lowest 20%. Panel B shows partial rank correlation coefficients, the correlation between the rank of a parameter and the rank of the outcome adjusted for all other parameters [33]. A value of 1 would indicate perfect square: the simulation with the greatest value of the parameter having the greatest value of the outcome, the simulation with the second-highest parameter having the second-greatest outcome, etc. The 6 most influential parameters for each analysis are shown.

Figure 5 demonstrates 2 complementary methods for identifying influential parameters. Panel A shows a 1-way sensitivity analysis, comparing the primary outcome (reduction in TB incidence by 2030 with active case finding) in simulations that use the highest vs lowest quintiles of each input parameter. Panel B shows a multiway sensitivity analysis using partial rank correlation coefficients (PRCCs): the correlation between the ranked value of the outcome and the ranked value of each individual parameter, adjusted for all other parameters [33]. These 2 methods identify influential parameters that are similar but not identical. One highly influential parameter in both analyses is the TB transmission rate—reflecting the fact that case-finding interventions likely have the greatest impact in settings where TB incidence is driven by recent transmission rather than reactivation. The PRCC of 0.91 shows that the transmission rate is highly correlated to the outcome, and the high- vs low-quintiles analysis illustrates the magnitude of that effect (a 21% reduction in incidence in the lowest quintile of the transmission rate vs a 38% reduction in the highest quintile). These results imply that the effect of the hypothetical case-finding intervention depends heavily on the degree to which transmission drives TB incidence in India.

Such analyses help to identify parameters that most impact model results and give an idea of how much results might change if key parameter values changed. In general, sensitivity analyses should address gaps in data, explore drivers of study results, and identify which parameters or structural features strongly influence conclusions. They provide an opportunity for communicating the limitations and uncertainty inherent to any modeling study.

## SYNTHESIS AND COMMUNICATION OF STUDY DESIGN

All models are imperfect representations of reality and subject to uncertainty. The ability to quantify this uncertainty is a key strength of rigorous models, which employ a careful selection of model structure, prior distributions for input parameters, and a calibration process. Because the complexity of modeling studies can obscure such details, it is critically important that these design choices and their limitations be presented clearly for readers to evaluate.

We present a list of points for clinicians and decision-makers to consider when evaluating models of chronic infectious diseases. These points address whether models are data-driven, scientifically principled, and transparently communicated (Box 1).

## CONCLUSIONS

Mathematical modeling studies fill a unique role and can provide important insights into the epidemiology of “chronic” infectious diseases. Such models are complex and depend on at least 3 key elements: a model structure that sufficiently represents reality, input parameters that accurately reflect our knowledge of disease epidemiology, and a calibration process that matches model simulations to real-world observations insofar as they are known. These design elements are often obscure to nonexpert readers but can have substantial influence on model results and policy implications. To help clarify these considerations, we have developed a simple guide for decision-makers to evaluate models of chronic infectious disease. In doing so, we hope to facilitate the ongoing communication between modelers and policy makers that is critical if model results are to improve the health of populations.

**Box 1: A Guide to Evaluation of Mathematical Models of Chronic Infectious Diseases**

1.Model structure

 •Model structure contains all features that impact the research question.

 •Model structure avoids extraneous complexities that require additional assumptions.

2.Input parameters and prior distributions

 •Point estimates and sampling ranges reasonably reflect setting-specific data.

 •Prior distributions are clearly stated and use central tendencies when some values are more likely than others and broad or uniform distributions when the data are very uncertain.

 •Justification is given for the general approach to setting the shapes and ranges of prior distributions (for example, using similar relative ranges and shapes unless data strongly suggest otherwise).

 •Close attention is given to input parameters identified as influential by sensitivity analyses.

3.Model calibration

 •The model is calibrated to the highest-quality data available.

 •Calibration focuses on targets that have bearing on the research question at hand.

 •Model fit to all calibration targets is clearly displayed, and the closeness of fit to each target is reasonable (a poor fit to relevant targets raises concern about mis-specification of the model).

 •The likelihood function is clearly stated and reflects uncertainty in the calibration targets, including any potential correlation between repeated measurements.

4.Sensitivity analyses

 •Analyses highlight influential parameters and clearly communicate the magnitude of effect of each parameter.

 •Sensitivity analyses consider the availability and quality of the underlying data and whether the model is highly sensitive to highly uncertain inputs.

 •Analyses consider the effect of individual parameters in the context of other model parameters.

5.Communication of results

 •Estimates of uncertainty are clearly presented.

 •The sources of uncertainty are clearly stated, including parameter uncertainty, the calibration process, and (if relevant) uncertainty in model structure.

 •Important model assumptions are listed.

## Supplementary Material

ofx172_suppl_supplementary_material
