# Erratum

**DOI:** 10.1093/ofid/ofx206

**Published:** 2018-01-25

“Mathematical Modeling of ‘Chronic’ Infectious Diseases: Unpacking the Black Box” by Fojo et al. [Open Forum Infect Dis 2017; 4(4)] is an Open Access article and should have contained the following copyright line: © The Author [2018]. Published by Oxford University Press [on behalf of Infectious Diseases Society of America]. This is an Open Access article distributed under the terms of the Creative Commons Attribution License (http://creativecommons.org/licenses/by/4.0/), which permits unrestricted reuse, distribution, and reproduction in any medium, provided the original work is properly cited.

The authors regret the error.

