# Supplementary material for: Mathematical Modeling of “Chronic” Infectious Diseases: Unpacking the Black Box
Source: Open Forum Infect Dis. 2017 Aug 14;4(4):ofx172. doi: 10.1093/ofid/ofx172 (PMC5716064; doi:10.1093/ofid/ofx172)
Supplement: ofx172_suppl_supplementary_material [file ofx172_suppl_supplementary_material.docx]

Contents

[**MODEL PARAMETERS** 2](#_Toc484617043)

[**THE MODEL FORM** 3](#_Toc484617044)

[**Differential Equations for Model 1:** 4](#_Toc484617045)

[**Differential Equations for Model 2:** 5](#_Toc484617046)

[**Differential Equations for Model 3:** 5](#_Toc484617047)

[**A BAYESIAN APPROACH TO MODELS OF INFECTIOUS DISEASES** 7](#_Toc484617048)

[**PRIOR DISTRIBUTIONS** 8](#_Toc484617049)

[**LIKELIHOOD FUNCTIONS** 9](#_Toc484617050)

[**Uniform Likelihood:** 9](#_Toc484617051)

[**Independent Normal Likelihood:** 9](#_Toc484617052)

[**Correlated Error Normal Likelihood:** 10](#_Toc484617053)

[**Binomial Likelihood:** 10](#_Toc484617054)

[**Joint Normal-Binomial Likelihood:** 10](#_Toc484617055)

[**REFERENCES** 12](#_Toc484617056)

# **MODEL PARAMETERS**

Table S1: Input Parameters and Sampling Ranges for Illustrative TB Model

| **Parameter** | **Estimate** | **Sampling Range^a^** | **Source** |
| --- | --- | --- | --- |
| Proportion of infections resulting in primary progression to active TB | 0.140 | 0.105 – 0.175^b^ | ^1^ |
| Reactivation rate of latent TB, <5 years from infection | 0.00485 / year | 0.00364 – 0.00606 / year ^b^ | ^2^ |
| Reactivation rate of latent TB, >5 years from infection | 0.0005 / year | 0.00024 – 0.00089 / year | ^3^ |
| Transmission rate in 2002 | 12 / year | 0 – 25 | Determined from initial simulations |
| Transmission rate in 2030 | 9 / year | 0 – 20 (constrained to be less than 2030 rate) | Determined from initial simulations |
| Duration of Infectiousness of active TB^c^ | 14.3 months | 7.6 to 26.1 months | Derived from 2015 WHO Estimates^4^ |
| Treatment success proportion among those never treated for TB | 0.74 | 0.56 – 0.93^b^ | Derived from 2016 WHO Estimates^5^ |
| Treatment success proportion among those previously treated for TB | 0.65 | 0.49 – treatment success proportion in never treated ^b^ | Derived from 2016 WHO Estimates^5^ |
| Relapse risk after successful treatment | 0.040 | 0.026 – 0.060 | ^6, 7^ |
| Relapse rate (among those who will relapse) | 1.50 / year | 1.125 to 1.875 / year^b^ | ^6^ |
| Excess mortality rate from active TB | 0.15 / year | 0.08 – 0.3 / year | ^8^ |
| Rate of spontaneous resolution of active TB | 0.13 / year | 0.09 – 0.20 / year | ^8^ |
| Relative risk of new infection for those with prior infection (compared to never infected) | 0.5 | 0.1 – 0.9 | ^1, 9^ |

^a^For uniform distributions, the sampling range is taken to be the bounds of the distribution. For log-normal or logit-normal distributions, we choose the distribution’s parameters (mean and standard deviation) such that the sampling range corresponds to a 95% uncertainty interval. ^b^For parameters where the literature only yields a point estimate, we use ±25% from the literature-supported estimate. ^c^The parameter used in the model is the rate of treatment initiation, which is the reciprocal of the duration of infectiousness minus the mortality and spontaneous resolution rates.

# **THE MODEL FORM**

In general, our discussions above apply to any model that maps input parameters to outputs. In models of infectious diseases, models that are dynamic – meaning that the state of a disease epidemic in the future depends on the state of the epidemic in the present – are usually most appropriate.^10, 11^

The particular models we use in this manuscript are compartmental models,^12^ which reduce to sets of differential equations which are presented below. Of note, these are deterministic models: one set of input parameters always produces exactly the same set of outputs. While some randomness in the model may help to reflect uncertainty, this is arguably less of an advantage in slow-moving, chronic infectious disease epidemics.

We solve the differential equations using the *deSolve* package in R. The code used to run the models is available at: https://github.com/tfojo13/ModelingChronicInfectiousDiseases.

## **Differential Equations for Model 1:**

| **Notation:**  • $U(t)$ = Uninfected  • $L(t)$ = Latently infected  • $A(t)$ = Active TB  • $P(t)$ = The total population [i.e. $P(t)=U(t)+L(t)+A(t)$]  • $\lambda$ = Transmission rate of TB  • $\rho$ = Reactivation rate of latent TB  • $\gamma$ = The risk of rapid progression to active TB  • $\alpha$ = The birth rate  • $\omega$ = The general population mortality rate  • $\mu$ = Excess mortality rate from TB  • $\beta$ = Rate of spontaneous resolution of active TB  • $\theta$ = Rate of treatment initiation  • $\pi$ = Proportion of those treated who are cured  • $\kappa$ = Latent protection factor (risk of new TB infection in someone who has been previously infected with TB relative to someone who has never been infected)  • $f(t)$ = The force of infection [i.e. $f(t)=\lambda\times A(t)/P(t)$] $dU(t)/dt=\alpha\times P(t)+\theta\times\pi\times A(t)-U(t)\times[f(t)+\omega]$   The uninfected increase through birth and active cases being treated and cured, and decrease by becoming infected with TB or dying. $dL(t)/dt=U(t)\times(1-\gamma)\times f(t)+A(t)\times\beta-L(t)\times[]\rho+\kappa\times\gamma\times f(t)+\omega]$   Those latently infected increase by uninfected becoming infected and active cases spontaneously resolving, and decrease by reactivating to active TB, becoming newly infected (again) with TB and rapidly progressing to active disease, and dying. $dA(t)/dt=[U(t)+\kappa\times L(t)]\times\gamma\times f(t)+L(t)\times\rho-A(t)\times[\theta\times\pi+\beta+\omega+\mu]$   Those with active TB increase by uninfected individuals becoming infected and rapidly progressing to active TB or by latently infected individuals becoming newly infected and rapidly progressing, and by latently infected individuals reactivating. They decrease by successful treatment, spontaneous resolution, and death (both overall and TB-specific mortality). |
| --- |

## **Differential Equations for Model 2:**

| Notation is as for model 1 with the following exceptions:  • $L_{1}(t)$ = Latently infected, with infection acquired less than 5 years ago  • $L_{2}(t)$ = Latently infected, with infection acquired more than 5 years ago  • $P(t)$ = The total population [i.e. $P(t)=U(t)+L_{1}(t)+L_{2}(t)+A(t)$]  • $\rho_{1}$ = Reactivation rate of latent TB, if infection was acquired less than 5 years ago  • $\rho_{2}$ = Reactivation rate of latent TB, if infection was acquired more than 5 years ago $dU(t)/dt=\alpha\times P(t)+\theta\times\pi\times A(t)-U(t)\times[f(t)+\omega]$   The uninfected increase through birth and active cases being treated and cured, and decrease by becoming infected with TB or dying. $dL_{1}(t)/dt=[U(t)+\kappa\times L_{2}(t)]\times(1-\gamma)\times f(t)+A(t)\times\beta-L_{1}(t)\times[\rho_{1}+1/5+\kappa\times\gamma\times f(t)+\omega]$   Those in the first latent state (fewer than 5 years from infection) increase by uninfected becoming infected or by those in the second latent state (more than 5 years from infection) acquiring a new latent infection, and by active cases spontaneously resolving. They decrease by reactivating to active TB, moving into the next latent state (at a rate of 1/5 per year), becoming newly infected (again) with TB and rapidly progressing to active disease, and dying. $dL_{2}(t)/dt=L_{1}(t)/5-L_{2}(t)\times[\rho_{2}+1/5+\kappa\times\gamma\times f(t)+\omega]$   Those in the second latent state (more than 5 years from infection) increase by individuals in the first latent state moving into the second latent state with the passage of time (at a rate of 1/5 per year). They decrease by reactivating to active TB, moving into the next latent state (at a rate of 1/5 per year), becoming newly infected (again) with TB and rapidly progressing to active disease, and dying. $dA(t)/dt=[U(t)+\kappa\times L_{1}(t)+\kappa\times L_{2}(t)]\times\gamma\times f(t)+L_{1}(t)\times\rho_{1}+L_{2}(t)\times\rho_{2}-A(t)\times[\theta\times\pi+\beta+\omega+\mu]$   Those with active TB increase by uninfected individuals becoming infected and rapidly progressing to active TB or by latently infected individuals becoming newly infected and rapidly progressing, and by latently infected individuals reactivating. They decrease by successful treatment, spontaneous resolution, and death (both overall and TB-specific mortality). |
| --- |

##

## **Differential Equations for Model 3:**

| Notation is as for model 2 with the following exceptions:  • n = a subscript indicating never before treated for TB  • p = a subscript indicating previously treated for TB  • $U_{n}(t),U_{p}(t)$ = Uninfected  • $L_{n,1}(t),L_{p,1}(t)$ = Latently infected, with infection acquired less than 5 years ago  • $L_{n,2}(t),L_{p,2}(t)$ = Latently infected, with infection acquired more than 5 years ago  • $A_{n}(t),A_{p}(t)$ = Active TB  • $R(t)$ = Those who have been treated for active TB and cured but will relapse  • $P(t)$ = The total population [i.e. $P(t)=U_{n}(t)+U_{p}(t)+L_{n,1}(t)+L_{p,1}(t)+L_{n,2}(t)+L_{p,2}t+A_{n}(t)+A_{p}(t)+R(t)$]  • $f(t)$ = The force of infection [i.e. $f(t)=\lambda\times[A_{n}(t)+A_{p}(t)]/P(t)$]  • $\pi_{n},\pi_{p}$ = Proportion of those treated who are cured  • $\tau$ = Proportion of those who are treated and cured who will eventually relapse  • $\varphi$ = Relapse rate, among those who will relapse $dU_{n}(t)/dt=\alpha\times P(t)-U_{n}(t)\times[f(t)+\omega]$   Those who are uninfected and have never been treated increase by birth and decrease by becoming infected with TB or dying. $dU_{p}(t)/dt=(1-\tau)\times\theta\times[\pi_{n}\times A_{n}(t)+\pi_{p}\times A_{p}(t)]-U_{p}(t)\times[\kappa\times f(t)+\omega]$   Those who are uninfected and have been previously treated for TB increase by active individuals being successfully treated and decrease by becoming infected with TB (again) or dying. $dL_{n,1}(t)/dt=[U_{n}(t)+\kappa\times L_{n,2}(t)]\times(1-\gamma)\times f(t)+A_{n}(t)\times\beta-L_{n,1}(t)\times[\rho_{1}+1/5+\kappa\times\gamma\times f(t)+\omega]$   Those in the first latent state (fewer than 5 years from infection) and never treated for TB increase by uninfected becoming infected or by those in the second latent state (more than 5 years from infection) acquiring a new latent infection, and by active cases spontaneously resolving. They decrease by reactivating to active TB, moving into the next latent state (at a rate of 1/5 per year), becoming newly infected (again) with TB and rapidly progressing to active disease, and dying. $dL_{p,1}(t)/dt=[U_{p}(t)+\kappa\times L_{p,2}(t)]\times(1-\gamma)\times f(t)+A_{p}(t)\times\beta-L_{p,1}(t)\times[\rho_{1}+1/5+\kappa\times\gamma\times f(t)+\omega]$   Those in the first latent state (fewer than 5 years from infection) and previously treated for TB increase by uninfected becoming infected or by those in the second latent state (more than 5 years from infection) acquiring a new latent infection, and by active cases spontaneously resolving. They decrease by reactivating to active TB, moving into the next latent state (at a rate of 1/5 per year), becoming newly infected (again) with TB and rapidly progressing to active disease, and dying. $dL_{n,2}(t)/dt=L_{n,1}(t)/5-L_{n,2}(t)\times[\rho_{2}+1/5+\kappa\times\gamma\times f(t)+\omega]$   Those in the second latent state (more than 5 years from infection) and never treated for TB increase by individuals in the first latent state moving into the second latent state with the passage of time (at a rate of 1/5 per year). They decrease by reactivating to active TB, moving into the next latent state (at a rate of 1/5 per year), becoming newly infected (again) with TB and rapidly progressing to active disease, and dying. $dL_{p,2}(t)/dt=L_{p,1}(t)/5-L_{p,2}(t)\times[\rho_{2}+1/5+\kappa\times\gamma\times f(t)+\omega]$   Those in the second latent state (more than 5 years from infection) and previously treated for TB increase by individuals in the first latent state moving into the second latent state with the passage of time (at a rate of 1/5 per year). They decrease by reactivating to active TB, moving into the next latent state (at a rate of 1/5 per year), becoming newly infected (again) with TB and rapidly progressing to active disease, and dying. $dA_{n}(t)/dt=[U_{n}(t)+\kappa\times L_{n,1}(t)+\kappa\times L_{n,2}(t)]\times\gamma\times f(t)+L_{n,1}(t)\times\rho_{1}$ $+L_{n,1}(t)\times\rho_{1}+L_{n,2}(t)\times\rho_{2}-A_{n}(t)\times[\theta+\beta+\omega+\mu]$   Those with active TB and never before treated for TB increase by uninfected individuals becoming infected and rapidly progressing to active TB or by latently infected individuals becoming newly infected and rapidly progressing, and by latently infected individuals reactivating. They decrease by starting treatment (if successful, they move to uninfected, if unsuccessful, they move to active TB but previously treated), spontaneous resolution, and death (both overall and TB-specific mortality). $dA_{p}(t)/dt=[U_{p}(t)+\kappa\times L_{p,1}(t)+\kappa\times L_{p,2}(t)]\times\gamma\times f(t)+L_{p,1}(t)\times\rho_{1}$ $+L_{p,2}(t)\times\rho_{2}+A_{n}(t)\times\theta\times(1-\pi_{n})+R(t)\times\varphi-A_{p}(t)\times[\theta\times\pi_{p}+\beta+\omega+\mu]$   Those with active TB and never before treated for TB increase by (1) uninfected individuals becoming infected and rapidly progressing to active TB or by latently infected individuals becoming newly infected and rapidly progressing, (2) by latently infected individuals reactivating, (3) by those with active TB who have never been treated failing their first course of therapy, and (4) those who have been successfully treated relapsing. They decrease by successful treatment, spontaneous resolution, and death (both overall and TB-specific mortality). $dR/dt=\tau\times[A_{n}(t)\times\theta\times\pi_{n}+A_{p}(t)\times\theta\times\pi_{p}]-R(t)\times[\varphi+\omega]$   Those who will ultimately relapse increase by the proportion of those with active TB who are treated and cured but will relapse, and are decreased by relapse or death. |
| --- |

# **A BAYESIAN APPROACH TO MODELS OF INFECTIOUS DISEASES**

In general, we can conceive of the model as a function that maps (possibly stochastically) input parameters to outputs. We can state this mathematically as M(**θ**) 🡪 **φ**, where M represents the model as a function, **θ** represents the input parameters, and **φ** represents the outputs. In practice, generating outputs from the input parameters involves non-trivial computations that we refer to as a simulation. In the compartmental model we use, M is a deterministic function; in other cases, such as agent-based models, M may be stochastic.

For a dynamic model of an infectious disease, the input parameters typically consist of disease-specific quantities such as transmission rate, treatment success, parameters governing latency, etc., as well as some non-disease related parameters like birth and death rates. The outputs will typically consist of metrics such as incidence, disease-specific mortality, proportion of infections that carry resistance, etc.

In a Bayesian framework, we specify prior distributions for the input parameters. We then apply a likelihood function that represents the probability that we would have observed the real-world data we have (such as disease incidence or mortality) if a particular model simulation were ‘the truth.’ While the likelihood can theoretically involve any of the input and output parameters, in practice, it generally deals only with a subset of the outputs. We can represent this mathematically as L(**y**; **φ**), where L is the likelihood function, **y** is our real-world data, and **φ** is the outputs.

Given prior distributions and a likelihood function, we can calculate a posterior distribution over all our parameters (both input parameters and output parameters). In our example, we used a Bayesian sampling/importance resampling approach,^13-15^ in which we (1) sample a large number of sets of input parameters according to their prior distributions, (2) run the model for each set of inputs to generate outputs, (3) resample simulations with replacement, where each simulation has a probability of being resamples proportional to its likelihood. This yields a large set of parameters values, [**θ**^(s)^, **φ**^(s)^], for s = 1, …, n (where n is the number of resamples). This set of parameter values approximates the posterior distribution of all parameters [**θ** , **φ**].

We can also approximate the posterior distribution using Markov chain Monte Carlo (MCMC) methods (we usually use Metropolis-Hastings).^16, 17^ MCMC methods have the advantage of being more efficient – i.e., requiring fewer overall simulations to approximate the posterior distribution well. However, the simulations must be run in series. Sampling/resampling algorithms (a Monte Carlo approach) have the advantage that simulations can be run in parallel, which can result in an overall time savings (at the cost of increased computation time) if running the model is very time intensive.

Regardless of the method used, once we have a posterior distribution over the input parameters and, consequently, the outputs, we can report summary statistics (for example, posterior mean and credible interval) over results of interest. The results we are interested can be a subset of the outputs – for example, to answer a research question about future incidence of disease. They can also include a subset of the input parameters – for example, to answer a research question about what transmission rates are compatible with the observed data from a disease epidemic.

Of note, there are alternative methods that fall under the umbrella of Approximate Bayesian Computation, which approximate a Bayesian solution without having to calculate the full likelihood.^18, 19^ These methods are particularly useful when explicitly solving for the likelihood is computationally intensive.

## **PRIOR DISTRIBUTIONS**

In general, most input parameters for dynamic models of the sort we are studying are either rates that can take values from zero to infinity (such as transmission rates, death rates, rates of treatment, etc.) or proportions that can take values from zero to one (risk of relapse, probability of drug resistance, etc.). While it may be appropriate to represent some parameters as a single number (for example, the mortality rate if vital statistics are highly accurate for a particular study setting), most input parameters are not exactly known. In such cases, we represent the uncertainty around a parameter by specifying a prior distribution.

In specifying a prior distribution, we have to decide (1) what particular probability distribution the input parameter will follow, and (2) what parameters to use for that distribution (such as the mean and standard deviation for a log-normal distribution). Often, a review of the literature will yield a “best-guess” estimate for a parameter or a range of estimates, but provides little data to support a particular distribution. We typically choose a distribution according to the values that an input parameter could theoretically take. For rates, which can range from zero to infinity, either a uniform distribution or a log-normal distribution produce appropriately-bounded values. For a proportion, a uniform distribution, logit-normal distribution, or beta distribution will produce values between zero and one. For both rates and proportions, using a normal distribution could conceivably yield values less than zero which are theoretically impossible.

In addition to choosing a form for a given parameter’s prior distribution, we also have to map the range of possible values drawn from the literature into appropriate parameters for that distribution. For a uniform distribution, it is simple to take the lowest and highest estimates from the literature as the lower and upper bounds of the distribution. For other distributions, we choose the parameters such that the lowest and highest estimates from the literature correspond to a 95% confidence interval.

When there are a large number of parameters, picking and choosing a distribution and range for each one can open the door to arbitrary choices of prior distributions that yield favorable results. For this reason, we advocate taking a principled approach to choosing prior distributions: deciding *a priori* to use (1) the same type of distribution for all rates and the same type of distribution for all proportions, (2) a fixed range of ±25% or ±50% from the best-guess estimate, except where the published literature strongly supports a specific range.

Lastly, we generally assume that, *a priori*, parameters are uncorrelated (i.e., have a covariance of zero). However, if we have strong prior information to the contrary, we can also sample from a joint prior distribution over several parameters with any arbitrary correlation structure. In our motivating example, the treatment success for those who have been previously treated for TB is constrained to be greater than the treatment success for those who are treatment-naïve, so the retreatment success proportion is sampled from a truncated distribution, with a lower bound at the success proportion for the treatment-naïve.

## **LIKELIHOOD FUNCTIONS**

Below, we outline the formulas behind several possible formulations of the likelihood function. In general, we will presume that the data for our likelihood consists of:

**y** = Our real-world data, the vector [y_1_, y_2_, …, y_T_]’

where y_t_ = some measurement that is repeated at multiple time points t, for t = 1, …, T. For example, we might have disease incidence across several years.

L_t_, U_t_ = the upper and lower bounds of some uncertainty interval around each measurement y_t._

Additionally, we let **µ** = the vector [µ_1_, µ_2_, …, µ_T_]’, where µ_t_ = the estimate of y_t_ according to the model simulation.

Among the likelihoods described below, the binomial constrains the mean-variance relationship, and for large sample sizes, allows for relatively little variation. It is generally most appropriate in settings where incidence and case numbers are low, and nearly all cases are detected. The likelihoods based on normal and uniform distributions allow for the variance to be specified independent of the mean, and thus make more sense when incidence is less certain and estimated from other observations (for example, incidence derived from prevalence survey results combined with an estimated average disease duration, or from a case notification rate combined with a large but uncertain rate of under-reporting, as in India and other high-TB-burden settings).

### **Uniform Likelihood:**

For one time point, we generate a uniform likelihood by assuming that the error, y_t_ - µ_t_ ~ Uniform(y_t_ - U_t_, y_t_ - L_t_):

L(y_t_, L_t_, U_t_; µ_t_) = **1**[y_t_ - U_t_ ≤ y_t_ - µ_t_ ≤ y_t_ - L_t_] = **1**[L_t_ ≤ µ_t_ ≤ U_t_]

Where **1**[…] denotes the indicator function. This likelihood is equivalent to testing whether the model estimate, µ_t_, falls within the uncertainty interval for the real-world data, [L_t_, U_t_].

The likelihood across all time points is then the product of the likelihoods for each year – i.e. 1 if all estimates fall within their uncertainty ranges versus 0 if any estimate falls outside its uncertainty range.

### **Independent Normal Likelihood:**

The uniform distribution has poor discrimination; simulations where all estimates are within uncertainty ranges are all equally likely, while any simulation with an estimate outside of the uncertainty ranges has zero likelihood. A normal likelihood offers a way to differentially weight simulations by how close estimates are to the real-world observations.

We generate the likelihood for one time point by assuming that

y_t_ ~ Normal(µ_t_, σ_t_^2^)

where we choose σ_t_^2^ such that [L_t_, U_t_] represents the 95% confidence interval of a normal distribution with mean y_t_ and standard deviation σ_t_.

The likelihood for all years t = 1, …, T is the product of the likelihood for each individual year, which makes the error for any one year, y_t_ - µ_t_, independent of the error for any other year. We could alternatively formulate this by saying

**y** ~ Multivariate Normal(**µ**, **Σ**)

where **Σ** is a TxT diagonal matrix such that **Σ**_tt_ = σ_t_^2^.

Under a normal likelihood, the differences between real-world observations and model estimates, y_t_ - µ_t_, principally represent potential measurement error in the method used to generate the observations **y** (for example, the way in which the WHO generates estimates of TB incidence).

### **Correlated Error Normal Likelihood:**

In reality, when the same method is used to generate estimates of disease (i.e. **y**) over multiple years, it is likely that measurement errors are correlated from year to year. To account for this correlation, we can generate a likelihood by assuming

**y** ~ Multivariate Normal(**µ**, **Σ**)

but explicitly specifying a correlation structure in our covariance matrix **Σ**.

To do this, we let

**Σ**_ij_ = σ_i_^2^ if i=j

**Σ**_ij_ = σ_i_ × σ_j_ × ρ_ij_ if i≠j

where ρ_ij_ represents the correlation between the errors at times i and j (i.e. the correlation between y_i_ - µ_i_ and y_j_ - µ_j_). We borrow from the techniques of generalized estimating equations (GEEs) in specifying the ρ_ij_.^20^ We favor using a compound symmetry structure, i.e., ρ_ij_ = ρ, where ρ is the same for all i,j when i≠j. This implies that the method used to generate estimates carries some error that is largely consistent across time. We could alternatively use an autoregressive structure, i.e. ρ_ij_ = ρ^|(time of i) - time of j)|^, which implies that measurement errors that are close to each other in time are closely correlated, while those farther apart in time are weakly correlated. Of note, choosing ρ_ij_ = 0 is mathematically equivalent to the independent normal likelihood described above. To our knowledge, at this time there are no general ways to estimate the value of ρ; in this analysis, we have assumed a compound symmetry structure with ρ = 0.5 as a compromise between complete independence (ρ = 0) and perfect correlation (ρ = 1).

### **Binomial Likelihood:**

In some settings, there is little to no uncertainty about real-world observations (settings where disease reporting is essentially complete). In such situations, a normal distribution with a variance related to the chance of measurement errors makes little sense. Instead, we prefer to use a binomial likelihood:

y_t_ х N_t_ ~ Binomial(N_t_, µ_t_)

In the case where y_t_ is the observed incidence, N_t_ represents the size of the population at risk for infection at time point t, y_t_ х N_t_ represents the number of individuals (a count) who became infected, and µ_t_ is the simulation-estimated incidence.

Under this likelihood, the differences between real-world observations and model estimates, y_t_ - µ_t_, principally represent the concept that the model is an imperfect representation of reality (i.e. that there is stochastic variation around our estimates of the mean). The likelihood for all data is the product of the binomial likelihood from each year. This makes the assumption that the errors, y_t_ х N_t_ - µ_t_ х N_t_, are conditionally independent given the probabilities of infection, µ_t_, for each year. As the errors in this formulation do not represent measurement errors, we find this to be a reasonable assumption.

### **Joint Normal-Binomial Likelihood:**

As a normal likelihood principally reflects measurement errors in the process used to generate real-world observations and the binomial likelihood reflects stochastic variation, we can combine these into one likelihood:

**y** | **µ*** ~ Multivariate Normal(**µ***, **Σ**)

where **Σ** is as defined for the normal likelihoods, and **µ*** is the vector [µ*_1_, µ*_2_, …, µ*_T_]’, such that,

µ*_t_ х N_t_ ~ Binomial(N_t_, µ_t_)

Using the normal approximation of the binomial, we can write:

µ*_t_ х N_t_ ~ Normal(µ_t_ х N_t_, µ_t_ х [1 - µ_t_] х N_t_)

µ*_t_ ~ Normal(µ_t_, µ_t_ х [1 - µ_t_])

**µ*** ~ Multivariate Normal(**µ**, **Σ***)

where **Σ*** is the diagonal matrix whose diagonal elements are µ_1_ х [1 - µ_1_], …, µ_T_ х [1 - µ_T_]. Then,

**y** ~ Multivariate Normal(**µ**, **Σ + Σ***)

In practice, the component of the variance from the binomial, **Σ*** (due to model imperfections), is usually very small relative to the variance from the initial multivariate normal component, **Σ** (due to measurement errors), so we ignore it and just use a normal likelihood as described above.

# **REFERENCES**

1. Vynnycky E, Fine PE. The natural history of tuberculosis: the implications of age-dependent risks of disease and the role of reinfection. *Epidemiol Infect*. 1997;**119**(2):183-201.

2. Sloot R, Schim van der Loeff MF, Kouw PM, Borgdorff MW. Risk of tuberculosis after recent exposure. A 10-year follow-up study of contacts in Amsterdam. *Am J Respir Crit Care Med*. 2014;**190**(9):1044-52.

3. Horsburgh CR, Jr., O'Donnell M, Chamblee S, Moreland JL, Johnson J, Marsh BJ, et al. Revisiting rates of reactivation tuberculosis: a population-based approach. *Am J Respir Crit Care Med*. 2010;**182**(3):420-5.

4. World Health Organization. Global tuberculosis report 2015. Geneva, Switzerland: World Health Organization; 2015.

5. World Health Organization. Global tuberculosis report 2016. Geneva, Switzerland: World Health Organization; 2016.

6. Marx FM, Dunbar R, Enarson DA, Williams BG, Warren RM, van der Spuy GD, et al. The temporal dynamics of relapse and reinfection tuberculosis after successful treatment: a retrospective cohort study. *Clin Infect Dis*. 2014;**58**(12):1676-83.

7. Menzies D, Benedetti A, Paydar A, Martin I, Royce S, Pai M, et al. Effect of duration and intermittency of rifampin on tuberculosis treatment outcomes: a systematic review and meta-analysis. *PLoS Med*. 2009;**6**(9):e1000146.

8. Tiemersma EW, van der Werf MJ, Borgdorff MW, Williams BG, Nagelkerke NJ. Natural history of tuberculosis: duration and fatality of untreated pulmonary tuberculosis in HIV negative patients: a systematic review. *PLoS One*. 2011;**6**(4):e17601.

9. Andrews JR, Noubary F, Walensky RP, Cerda R, Losina E, Horsburgh CR. Risk of progression to active tuberculosis following reinfection with Mycobacterium tuberculosis. *Clin Infect Dis*. 2012;**54**(6):784-91.

10. Pitman R, Fisman D, Zaric GS, Postma M, Kretzschmar M, Edmunds J, et al. Dynamic transmission modeling: a report of the ISPOR-SMDM Modeling Good Research Practices Task Force Working Group-5. *Med Decis Making*. 2012;**32**(5):712-21.

11. Anderson RM, May RM. Infectious diseases of humans : dynamics and control. Oxford ; New York: Oxford University Press; 1991. viii, 757 p. p.

12. Brauer F, Van den Driessche P, Wu J, Allen LJS. Mathematical epidemiology. Berlin: Springer; 2008. xviii, 408 p. p.

13. Poole D, Raftery AE. Inference for Deterministic Simulation Models: The Bayesian Melding Approach. *Journal of the American Statistical Association*. 2000;**95**(452):1244.

14. Alkema L, Raftery AE, Clark SJ. Probabilistic projections of HIV prevalence using Bayesian melding. *The Annals of Applied Statistics*. 2007;**1**(1):229-48.

15. Alkema L, Raftery AE, Brown T. Bayesian melding for estimating uncertainty in national HIV prevalence estimates. *Sexually Transmitted Infections*. 2008;**84**(Supplement 1):i11-i6.

16. Berg BA. Markov chain Monte Carlo simulations and their statistical analysis : with web-based Fortran code. Hackensack, NJ: World Scientific; 2004. xv, 361 p. p.

17. Hoff PD. A first course in Bayesian statistical methods. London ; New York: Springer; 2009. ix, 270 p. p.

18. van der Vaart E, Beaumont MA, Johnston ASA, Sibly RM. Calibration and evaluation of individual-based models using Approximate Bayesian Computation. *Ecological Modelling*. 2015;**312**:182-90.

19. Yates A, Coelho FC, Codeço CT, Gomes MGM. A Bayesian Framework for Parameter Estimation in Dynamical Models. *PLoS ONE*. 2011;**6**(5):e19616.

20. Zeger SL, Liang KY. Longitudinal data analysis for discrete and continuous outcomes. *Biometrics*. 1986;**42**(1):121-30.
